# A Hypergraph-Based Blockchain Model and Application in Internet of Things-Enabled Smart Homes

**DOI:** 10.3390/s18092784

**Published:** 2018-08-24

**Authors:** Chao Qu, Ming Tao, Ruifen Yuan

**Affiliations:** School of Computer Science and Network Security, Dongguan University of Technology, Dongguan 523808, China; quc@dgut.edu.cn (C.Q.); yuanrf@dgut.edu.cn (R.Y.)

**Keywords:** hypergraph, blockchain, IoT, smart home

## Abstract

With the fast development and expansion of the Internet of Things (IoT), billions of smart devices are being continuously connected, and smart homes, as a typical IoT application, are providing people with various convenient applications, but face security and privacy issues. The idea of Blockchain (BC) theory has brought about a potential solution to the IoT security problem. The emergence of blockchain technology has brought about a change of decentralized management, providing an effective solution for the protection of network security and privacy. On the other hand, the smart devices in IoT are always lightweight and have less energy and memory. This makes the application of blockchain difficult. Against this background, this paper proposes a blockchain model based on hypergraphs. The aims of this model are to reduce the storage consumption and to solve the additional security issues. In the model, we use the hyperedge as the organization of storage nodes and convert the entire networked data storage into part network storage. We discuss the design of the model and security strategy in detail, introducing some use cases in a smart home network and evaluating the storage performance of the model through simulation experiments and an evaluation of the network.

## 1. Introduction

The Internet of things (IoT) is a worldwide network of interconnected objects and humans, which through unique address schemes are able to interact with each other and cooperate with their neighbours to reach common goals. The primary purpose of the IoT is to share information acquired by objects, which reflects the manufacture, transportation, consumption and other details of people’s lives [1,2]. Therefore, most of these new networkable devices are designed to be lightweight and have less memory [3]. Due to the low-cost price of processors and wireless cards, almost anything can be part of the IoT, from wearable devices [4] (such as smart wrist straps and smart watches) to a giant transportation vehicle (such as a train or airplane) [5]. A Gartner analysis reported that 8.4 billion connected IoT units were used worldwide in 2017, up 31% from 2016, and this number is expected to expand to 20.4 billion by 2020. Total spending on endpoints and services reached almost 2 trillion dollars in 2017 with two-thirds of those devices found in China, North America and Western Europe. The development of the IoT has created a large number of devices, such as sensors, interconnected and interoperable devices for data collection and exchange. The data obtained from the IoT can make our life more convenient and comfortable through many applications.

The most popular application for IoT is the smart home, which offers a better quality of life by introducing automated appliance control and assistive services. IoT devices work collaboratively and optimize user comfort by using context awareness and predefined constraints based on the conditions of the home environment. Smart homes provide comfort and security services to their inhabitants [6]. Of these, the most important is security, which not only provides authentication services to the user but also restricts unauthorized access to the household’s devices [7]. As more and more personal information is collected and communicated in the smart home network (and possibly with other wired and wireless networks), security and privacy issues have become more pronounced and must be seriously taken into account in order to exploit the full benefits of smart home environments. Today, smart home security is an important area of research, with many theories and methodologies being proposed [8,9,10]. Dorri et al. [11] proposed that each smart home is equipped with an always online, high resource “miner” that is responsible for handling all communication within and external to the home. Bertino [12] outlined key challenges in data security and privacy and summarize research directions for securing IoT data. Shafagh et al. [13] enabled a secure and persistent data management, by utilizing the blockchain as an auditable access control layer to a decentralized storage layer. In this paper we focus on the reduction of storage capacity for IoT devices and the security and privacy protection of data streams in smart home systems. Cyber-security in a smart home requires the protection of the IoT data flow from malicious activity, which can require a costly budget and a long process.

Blockchain technology offers one way to enhance smart home security by using the ledger of data transmitted from one home to another home and prevent the abnormal communications. Blockchain is a decentralized network which enables all parties to make transactions in a trustless network. The blockchain approach has been widely applied to many fields such as finance [14], insurance [15], manufacturing [16], and healthcare [17]. The blockchain has a distributed ledger that contains connected blocks of transactions for all the members in a network. The security and privacy issues of smart homes may be solvable with blockchain technology. Kshetri [18], demonstrated that blockchain-based identity and access management systems have the ability to significantly strengthen IoT security. Dorri [19], proposed a hierarchical architecture that uses a centralized private immutable ledger at the local IoT network level in a single smart home to decrease overhead and a decentralized public blockchain at higher-end devices for stronger trust.

Blockchain-based security and privacy protection can solve problems brought about by centralized management and provide better solutions. However, even so, the combination of blockchain and smart homes still has many problems. The issues and contributions of this paper are as follows:

1. We propose a hypergraph-based blockchain model. The implementation of blockchain technology requires that all nodes in the network maintain synchronized data records, which will undoubtedly put a lot of pressure on data storage. Therefore, to a certain extent, reducing the number of nodes that synchronize data in the network can also guarantee the normal operation of the blockchain. We use hypergraph theory to partition the entire network into many hyperedges, and each hyperedge stores a part of transaction data to reduce the storage pressure.
2. We discuss the additional security risks of the proposed model and put forward response strategies. The original blockchain technology is robust to single-point attacks. If a node is forged, the whole hash values in its blocks will be different from the others and it will be dropped from the network. If more than 51% of the nodes are forged, the false data alarm will take effect, but that is very difficult to implement in a network-scale environment [20]. In our model, the constraints are weakened, but we can reduce the risk to an acceptable level through the setting of network parameters, especially for IoT environments where security requirements are not very high.
3. We propose a dynamic network evolution algorithm. Considering the rapid expansion of IoT devices, IoT and smart home networks are growing at a geometric progression. When using hypergraph theory to partition blockchain networks, the algorithm takes the dynamic characteristics of the network into account, and due to the low power consumption and low processing capacity of IoT devices, the algorithm also needs to be designed relatively simply. An integer linear independence matrix is added to each node and each vector in the matrix map to a hyperedge. When the number of nodes increases or decreases, hyperedges can be easily split or aggregated according to the algorithm in order to guarantee the minimum cardinality of the graph.

The rest of the paper is organized as follow: Section 2 presents the related works. The hypergraph-based blockchain model is discussed in depth in Section 3. The use cases of smart homes are presented in Section 4. Section 5 analyses the experiments, and Section 6 concludes the paper.

## 2. Related Works

### 2.1. IoT and Smart Home

As an emerging application field that combines multiple technologies, IoT and smart homes combine to realize the intelligent management of the modern home environment by embedding various smart chips into home equipment. A smart home system is a typical ubiquitous computing environment, but there are a lot of problems that need to be solved. Ali [21], detected the security threats by posing several scenarios and evaluating the impact of these threats on a smart home environment. Chifor et al. [22] proposed a lightweight authorization stack for smart home IoT applications and the architecture is user-device centric. Khan [23] categorized popular security issues with regard to the IoT layered architecture, in addition to protocols used for networking, communication, and management. They point out that blockchain, which is the underlying technology for bitcoin, can be a key enabler to solve many IoT security problems. The recent research by Dorri [11,19] studied IoT and smart homes based on blockchain and presented a lightweight implementation of a BC particularly geared for use in IoT. In our previous work [24], a new blockchain architecture for smart homes was proposed and we explained the credibility verification method under the architecture.

### 2.2. Blockchain

**Figure 1:**
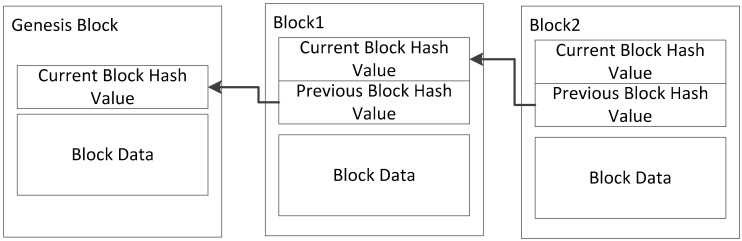
Blockchain.

Blockchain technology first came to prominence in early 2009 through the crypto-currency bitcoin (BTC). Bitcoin users use a variable public key (PK) to generate transaction information and broadcast it to the network for the transfer of funds. The transaction information is stored by all users in its own block. Once the block is full, a network mining process is performed; the block’s hash value is calculated, and the encrypted information and blocks are appended to the block chain. In order to mine the cryptographic hash value of a block, certain nodes in the network, known as miners, compete to solve a resource consumption cryptographic puzzle called proof of work (POW) [25]. The node that first solves the puzzle and gets everyone’s approval is considered to have tapped the block. BTC has flourished, and blockchain could, according to Swan [26], have far-ranging consequences for all aspects of modern society. This is because blockchain technology keeps all transaction data accounts through all members, and all members update the accounts simultaneously to maintain completeness when new transactions occur. Internet and encryption technologies are the underlying technologies that enable all members to verify the reliability of each transaction so that a single point of failure caused by a traditional third-party-authorized transaction is resolved. The blockchain has the characteristic of being broker-free (P2P-based), so the transaction eliminates unauthorized costs by the third party. Since everyone keeps the transaction information in sync, the modify records hacking effect of the single-point mode becomes very limited and often does not work. In addition, users of a blockchain system can openly access transaction records and reduce transaction supervision costs. Since the hash value stored in each peer in the block is affected by the value of the previous block, forging and changing data requires modification of the entire chain, and the amount of single-point calculation is far behind the calculation of the entire network. As a result, counterfeiting is almost impossible. Although data changes are possible if 51% of the calculations are involved in forgery, the record modify is very difficult to implement in a network-scale environment [20]. The blockchain network is constructed of many smart devices, and each device is considered to be a node. The blockchain node contains a data link as shown in Figure 1. Each data link includes many data blocks which contain the previous block hash and some transaction information as block data.

Based on the characteristics of blockchain, many researchers have examined its application in the IoT environment. Ouaddah et al. [25] proposed a framework for access control in IoT based on blockchain technology and provided a reference model for a proposed framework within the objectives, models, architecture and mechanism specification in IoT. Shen et al. [27] applied blockchain to a smart home system to ensure the security and privacy of information. Christidis et al. [28] applied a smart contract to the IoT, facilitating the sharing of services and resources and allowing automation in a cryptographically verifiable manner several existing time-consuming workflows. Huh et al. [29] used the blockchain platform to build an IoT system, to manage IoT devices and to control and configure IoT devices. Dorri et al. [19] proposed a lightweight blockchain-based architecture for IoT that virtually eliminates the overheads of classic blockchain, while maintaining most of its security and privacy benefits. Ouaddan et al. [30] implemented blockchain to smart device access and proposed “FairAccess” as a new decentralized pseudonymous and privacy preserving authorization management framework. Samaniego and Deters [31] presented the idea of using blockchain as a service for IoT and evaluated the performance of a cloud and edge hosted blockchain implementation. In [32], Raman and Varshney studied dynamic distributed storage for scaling blockchains in an IoT environment. The reason for this explosion of interest is that with blockchain technology in place, applications that previously could only be run through a trusted intermediary can now run in a decentralized manner.

### 2.3. Hypergraph

A hypergraph may be the most general concept in discrete mathematics, a system of finite sets and forms. Over the past few decades, hypergraph theory has been shown to helpful in solving real-world problems. As a mathematical tool, hypergraphs can be used to simulate computer networks, biological networks, data structures, process scheduling, and various other systems. The objects in the system and the complex relationships between them can often be mapped by hypergraphs, so as to seek an effective solution for various complex problems [33]. For applications in the IoT, Jung et al. proposed a hypergraph-based multidimensional structure to model the IoT for efficient management and discovery of IoT objects [34]. Yao et al. [35] developed a hypergraph to model objects’ spatiotemporal correlations.

**Figure 2:**
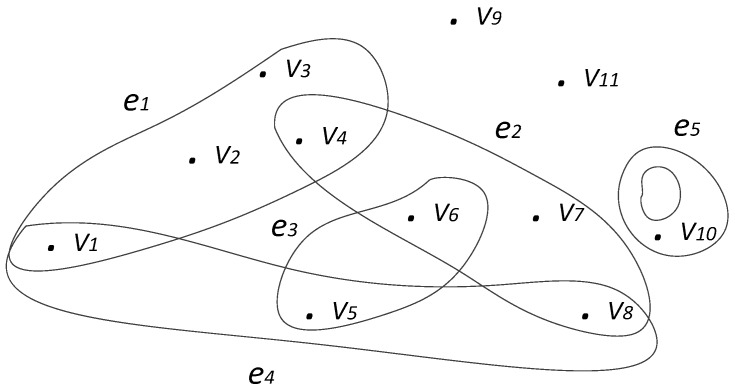
Example of a hypergraph.

A hypergraph *H* denoted by *H* = (*V*; *E*) on a finite set *V. E* = (*e_i_*)*_i__∈I_*, where *I* is a finite set of indexes, is a subset of *V* called a hyperedge. Commonly, *V* is a set of vertexes and denoted by *V*(*H*) and *E* by *E*(*H*). If two vertexes are in one hyperedge, they are called adjacent. The cardinality of a hyperedge denoted by |*e_i_*| is the count of vertexes in the hyperedge. The rank of *H* denoted by *r*(*H*) = *max_i__∈I_*|*e_i_*| is the maximum cardinality of hyperedges in the hypergraph; the minimum cardinality of hyperedges called the co-rank of *H* is denoted by *cr*(*H*) = *min_i__∈I_*|*e_i_*|. Two hyperedges in a hypergraph are adjacent if their intersection is not empty. The degree of a vertex is the count of hyperedges which include it. And the max degree is defined as the graph’s degree. An example of a hypergraph is shown in Figure 2.

Hypergraph *H* in Figure 2 has five hyperedges (*e*_1_ to *e*_5_) and 11 vertices (*V*_1_ to *V*_11_). *e*_5_ is a loop. The hypergraph contains two isolated vertices: *V*_9_, *V*_11_. The cardinalities of each hyperedge are *c*(*e*_1_) = 4, *c*(*e*_2_) = 4, *c*(*e*_3_) = 2, *c*(*e*_4_) = 3 and *c*(*e*_5_) = 1. The rank *r*(*H*) = 4 (contributed by *e*_1_ and *e*_2_), and the co-rank *cr*(*H*) = 1 (contributed by *e*_5_). The degree of *V*_1_ is 2 (because it is included by *e*_1_ and *e*_4_).

## 3. Hypergraph Based Blockchain Model

### 3.1. Problem Statement

In our opinion, the distributed ledger of the blockchain requires an all-network accounting mode in which all members keep a ledger containing all transaction data and update their ledgers to maintain integrity when there is a new transaction. Just as in a market, when a transaction occurs, the transaction party issues a statement, and then all the people check the correctness of the transaction and create a record. During the entire process, the information is transparent and uniform, and the participants’ qualifications and permissions are completely equal. The result confirmed by most people is the final conclusion, and the system will automatically correct the data for everyone’s approved results. Someone cannot cheat or insist on different views unless more than 51% of the people agree. When a great number of people involved to a certain extent, this is virtually impossible. However, the untrustworthiness of the transaction does not need 51% of the people in the market believe the transaction is illegal. The untrustworthiness threshold is often much lower. For example, if 10% of people think that the transaction is illegal, the transaction can be considered invalid.

Based on such a premise, for the transaction records in the entire blockchain network, it is not always necessary for all nodes to record them. For one transaction record, it is enough to make sure that a sufficient number of nodes record it. When another transaction occurs, the nodes that recorded the information related to it verify it. If the transaction is not legal, these nodes will send a signal that “the transaction is illegal”. When these signals reach a certain amount, the transaction is considered illegal; otherwise the transaction can be accepted. In such a mode of operation, the following issues must be addressed:

1. *The architecture of the model*. In order to implement the proposed scheme, a structure is needed to support the blockchain networks, and only certain nodes are used to store a transaction record without affecting security and privacy or reducing security risks to acceptable conditions.
2. *How the scheme works*. Under the designed structure, some mechanisms or algorithms are needed to support the structure to solve the problems caused by structural changes.
3. *The parameters set*. In the support structure and the algorithm designed for this, a series of parameters needs to be adjusted so that they can achieve the desired purpose in an optimal way. The setting of these parameters needs to be adjusted through experiments and evaluations.

### 3.2. Architecture Overview

**Figure 3:**
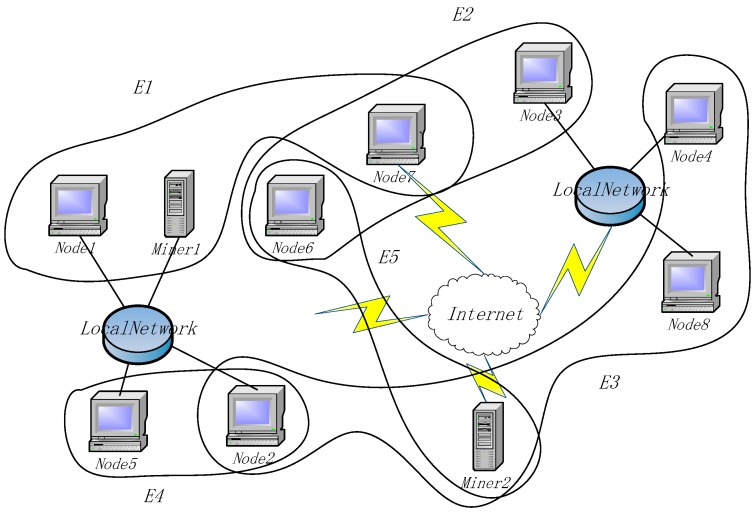
Hypergraph based Blockchain architecture.

In order to achieve the foregoing solution, we design an improved blockchain model. In the model, the nodes in the blockchain network are divided into several clusters, each with the same transaction record chain. At the same time, due to the dynamic nature of the blockchain network, such as in the IoT environment, it is not possible to formulate in a static manner which node clusters record which transaction records. For security and privacy considerations, the nodes that record the transactions appear in the blockchain network as anonymously as possible. The hypergraph theory provides us with a mathematical model of the structural design. According to the organizational structure of the hypergraph, the blockchain model we designed is shown in Figure 3.

In Figure 3, devices are randomly contained in five hyperedges, whether they are connected in the network or not. As shown in Figure 3, this simple blockchain network contains 10 nodes (named Node1 to Node10) and two miners (named Miner1 and Miner2). We take the nodes and miners as vertexes set *V*. There are five hyperedges in the network, *E1* to *E5*. As can be seen from Figure 3, the blockchain network is mapped to a hypergraph, where the devices are nodes in the graph, and each hyperedge corresponds to a set of devices. There are associated nodes between the hyperedges, that is, a device belongs to two or more hyperedges at the same time. For example, *Node6* in Figure 3 belongs to hyperedge *E2* and also belongs to hyperedge *E5*. Devices that belong to the same hyperedge may or may not be in the same local network, either as nodes or as miners. The objects in the architecture are explained as follows:

- *Nodes*: A node is a device with storage ability in a blockchain network. The route is reachable in the network and normal communication can be performed. Each node in the network belongs to at least one hyperedge and can belong to multiple hyperedges at the same time.
- *Miners*: Miners are devices for calculating encryption block hash keys in blockchain network, which is not much different from ordinary miners. However, in the designed architecture, miners also undertake the task of calculating the linear independence matrix (explained in Section 3.3.2) in the network, which is mainly used for the control of transaction data storage and network evolution.
- *Hyperedge*: A hyperedge is a set of nodes. All nodes on the same hyperedge have the same vector encoding that is independent of other hyperedges, and they have synchrony when storing transaction data.

### 3.3. Architecture Principal

#### 3.3.1. Network Parameters

In the designed architecture, nodes can belong to multiple hyperedges at the same time, and the nodes in the same hyperedge are synchronized in the transaction record storage process, which will lead to the simultaneous storage of nodes in multiple hyperedges. In order to make the storage distribution of transaction records more balanced and avoid the Matthew effect (i.e., some nodes in most hyperedges store large amounts of data, but others are just the opposite), the degree of each node in the specified network must be N, which can be regarded as a network parameter.

According to this idea, a transaction record is recorded by some nodes in the blockchain network. Therefore, in the designed architecture, a transaction record is recorded by nodes in a hyperedge. Considering that the number of nodes in the hyperedge may be unbalanced, the lower and upper limits of the node number denoted by co-rank and rank must be specified. Usually, a parameter C is set as the rank, and C/2 is the co-rank. Similarly, C can also be regarded as a network parameter.

Nodes newly added to the network should be added to N hyperedges at the same time. If the number of nodes on a hyperedge has reaches the upper limit, the hyperedge needs to split into two hyperedges with the number of nodes being C/2 and C/2+1.

#### 3.3.2. Node Blockchain

**Figure 4:**
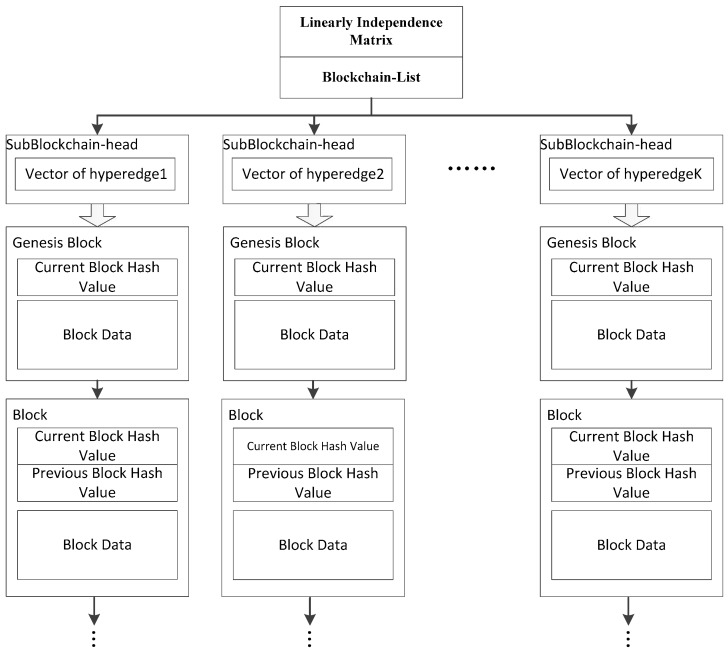
The data structure of node blocks.

Based on the proposed architecture, the whole working mechanism of the blockchain network is different from the original. Thus, the data structure of the node in the hypergraph and the storage function must be redesigned. Because a node will belong to multiple hyperedges at the same time, and the nodes in each hyperedge require storage synchronization, the nodes must also store transaction information synchronized in different hyperedges. In this case, if the original blockchain structure is adopted, the data blocks in the nodes will no longer be the same. When the current block is full and must be encrypted, the hash values calculated by each node are always different. This leads to the failure of the entire network. In order to counter this problem, we designed the data structure in each node as shown in Figure 4.

The structure of storage in each node is designed with two parts: the blockchain head and SubBlockchains. The blockchain head is constructed by a linear independence matrix, a vector and a Blockchain-list. The linear independence matrix is an N-order integer matrix consisting of N linearly independent vectors, each of which map to a hyperedge as its feature. N represents the number of hyperedges in the network. That means for each hyperedge in the blockchain network, there is an N-dimensional vector associated with it. The vector can be regarded as the ID of the hyperedge. The reason why we use a linear independence matrix is that when the network evolves, it is difficult to generate a new ID for a new hyperedge synchronously, but it is easy to generate a linear independence vector from a linear independence matrix.

**Figure 5:**
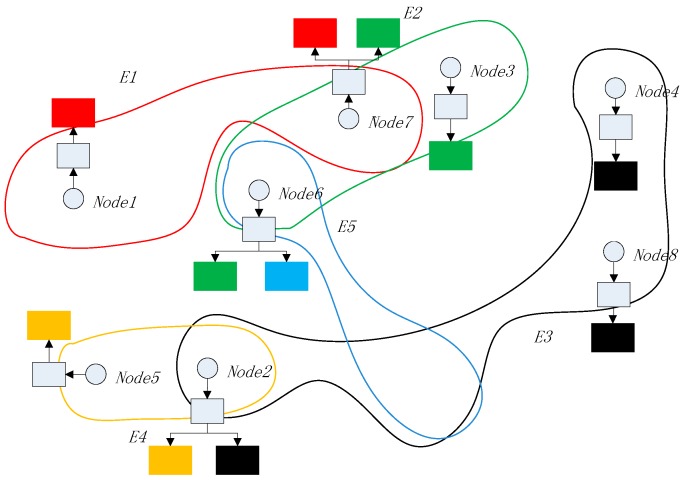
The data model of a hypergraph-based blockchain network.

The blockchain-list contains several indexes of blockchains. Each index points to a subblockchain. The count of subblockchains is the same as the degree of the node. A subblockchain is a kind of blockchain with a head, in which there is an N-dimensional vector as a hyperedge feature. Each of the sbublockchain stores synchronous transaction records separately in the hyperedge, whose feature vector is the same as the vector in the subblockchain head. Therefore, the nodes in the same hyperedge must have a same subblockchain. The data model of Figure 3 is explained in Figure 5.

From Figure 5, it can be seen that *Node1* is only included in *E1*; therefore, it only contains one subblockchain. *Node6* has two subblockchains because it is included in two hyperedges. Commonly, miners do not store data so they do not appear in Figure 5.

### 3.4. Working Mechanism

(A) Transaction publication

When a transaction occurs, the source node constructs a record that includes the following information: timestamp, the selected linearly independent vector (an integer N-dimensional vector) and the common information, such as the parties to the transaction, transaction content, needed in blockchain technology. What needs special attention is that the source node randomly finds a vector different from the linear independence vector in its own subblockchain head from the linear independence matrix. And add this vector to the record as the record feature.

(B) Transaction verification

First, when a transaction occurs, all nodes in the network receive the declaration and search for related records in their subblockchains. The nodes which stored the latest transaction information of the source node obtain the arbitration right. The arbitration nodes verify the declaration. If it is legal, then the arbitration nodes send a message that the transaction is legal to the network; otherwise, a message will be sent that the transaction is illegal. It is different from the original blockchain mechanism in which every node has the whole record and can verify the declaration by itself. In our model, the verification is done by some of the nodes, which send the result to others.

After getting the verification messages, the nodes in the network can judge the legitimacy of transactions based on the relationship of the average cardinality and the number of messages received, as well as the ratio between the legal certificate and the number of illegal certifications. Simply, for a given threshold, if the legal message count exceeds the threshold, the transaction is considered legal. The security of messages is guaranteed by a secret key system. For example, if a node gets 10 illegal messages and 90 legal messages and the threshold is 95% then the transaction will be considered illegal and will not be recorded. The security risks are discussed in Section 3.5.

(C) Record storage

When a transaction occurs, Nodes in the blockchain network compare the recorded feature vector with the vectors in its own subblockchain head. If matched and the transaction is verified to be legal, the record is added to the current block of corresponding subblockchain.

(D) Block encryption

When the current block in a certain subblockchain of a certain node is full (of course, the current block of the subblockchain corresponding to other nodes in the same hyperedge characterized by the vector in the subblockchain head is also full), according to blockchain working principle, the data of the current full block, previous block hash value and other information will be published to the network. All miners will receive these data and calculate an encryption hash value competitively. When a miner solves the puzzle, it will publish it to the network and the nodes which acquire this POW will verify the result easily. If the result is acceptable, the block will be encrypted and stored, otherwise the result will be dropped and the calculation continues. Comparing with the original blockchain model, the differences are shown in Table 1.

### 3.5. Security Discussion and Response Strategy

As mentioned in our model, records are stored separately, and almost no one has the copy of the whole recodes, that is different with distributed storage like [32] which uses a coding scheme to reduce storage capacity and ensure the whole record’s integrity in each node. This leads to the following additional security risks:

- Attacks on the storage nodes can be easier than in the original blockchain network;
- A verification attack will try to forge the legal message and increase the legal ratio;
- Attacks can occur from forging a new hyperedge and modifying the records only recorded by it.

Here we are not discussing the strategies of responding to the security problems of the original blockchain, such as the 51% calculation attack [20], DDoS attack [36] and mining attack [37]. The additional security risks are what must be faced and must be responded to.

For the first security risk, we stored records separately, and one record is not stored in all nodes but only in the nodes of one hyperedge. The attack in original blockchain must cover 51% nodes. But in our model, in order to make the attack take effect, the attacker only need cover 51% or more nodes in a hyperedge, the scope of the attack is greatly reduced. Based on the working mechanism, the success rate of this kind of attack depends on two factors: the co-rank (C/2) and the verification threshold. The co-rank determines at least how many nodes are in a hyperedge and the verification threshold determines how many of them are forged if the attack wants to be trusted. Therefore a higher co-rank and a higher verification threshold are recommended to protect against this security risk. Especially, if the co-rank equals N, which is the count of the nodes, this model evolves into the original blockchain.

For the second security risk, the secret key system provides the basis for protection against it. The arbitration message must be sent after encryption with a private key and the receiver uses the public key to decrypt the message. If an attacker wants to forge enough messages, he must control enough nodes in the blockchain network. The problem is also due to the co-rank and the verification threshold. For example, given co-rank = *c* and the threshold = *t* (*t* ∈ [0, 1]), when a forged transaction happens, at least *c* nodes think it is illegal. If the attacker wants the transaction to be trusted, he must control at least *c*/(1 − *t*) nodes. The higher *c* and *t*, the more nodes needed. High co-rank and verification threshold are recommended, also. But on the opposite side, if the threshold is too high, a denial of illegal attack will happen.

The main idea of dealing with the last security risk is to not let the forged nodes aggregate in one hyperedge. Instead, the newly added nodes are spread out as much as possible into the existing hyperedges. The cost of creating a forged hyperedge should be no easier than forging a record in the blockchain network. A solution algorithm is presented in Section 3.6 which is related to the graph’s co-rank.

### 3.6. Hyperedge Splitting and Aggregation

As we discussed, the number of nodes in each hyperedge must be guaranteed to be “enough” to meet the security requirements. In order to avoid the construction of a hyperedge with only a few nodes or a hyperedge only contains a few nodes when some nodes deleted from the network or prevent a forged hyperedge to be generated easier, we design an algorithm to manage the joining and deletion of nodes. The main idea is that when a new node joins the network, it is added to several hyperedges randomly, and if a hyperedge’s cardinality is over the rank of the graph, the hyperedge is separated into two, one with cardinality of *cr* and one with *cr*+*1*.

For a given rank *r*(*H*) the co-rank *cr*(*H*) is denoted as *r*(*H*)/2 and the Algorithm 1 for adding the node works as follows:


**Algorithm 1**
 Select a hyperedge *e_i_* randomly  if |*e_i_*| < *r*  insert the node *n* else  split the *r*/*2* of the nodes in *e_i_* to a new hyperedge *e_i_”*  insert the node *n* to *e_i_”* and copy the synchronous SubBlockchain  generate a new linear independence vector *V’* for *e_i_”*  for each node *n_i_* in the network add *V’* to the linear independence matrix

When a node is deleted from a network, it may cause a hyperedge’s cardinality to be less than *cr*, so the algorithm will work as follows: The cardinality of the hyperedge, denoted as *e_i_*, must be *cr*−*1*, and if there is a hyperedge *e_j_* with cardinality more than *cr* and not adjacent to *e_i_* then |*e*_j_|−*cr*(*H*) nodes are moved from *e_j_* to *e_i_*. If unfortunately, there is no such *e_j_* then a hyperedge (of course its cardinality is *cr*) is randomly selected and combined with *e_i_*. The deletion Algorithm 2 works as follows:


**Algorithm 2**
 for each hyperedge *e_i_* that contains the deleted node *n*
  if |*e_i_*| < *cr*  if exist a hyperedge *e_j_* and|*e_j_*| > *cr* and *e_i_*∩*e_j_* = Φ    for each *n_k_* (*k*∈[*cr,* |*e_j_*|]) in *e_j_* move *n_k_* to *e_i_* and copy the SubBlockchain of *e_i_*   else randomly select a hyperedge *e_k_* and combine it with *e_i_*

**Figure 6:**
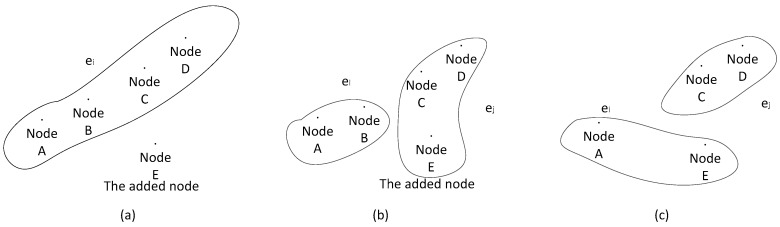
Adding a node and deleting a node from a network, (**a**) before Node E is added, (**b**) after Node E is added, (**c**) after Node B is deleted.

The algorithm guarantees that each transaction record is recorded by at least *r/2* nodes. When the hyperedge is split, it is equivalent to adding an extra hyperedge. When the hyperedges converge, it is equivalent to connecting two hyperedges. A schematic diagram of splitting and aggregation is shown in Figure 6 where *r* = 4. In order to keep the connection of hyperedges, when a node is added to the network, it is commonly added to several hyperedges simultaneously.

## 4. Use Case in a Smart Home

In this section, we apply the proposed hypergraph-based blockchain model to smart home system and discuss the model. The research of smart home systems is mainly divided into two categories: the interactions between devices within the home and the interactions among homes as independent nodes. Under the smart home environment, the in-house devices number is usually maintained at a lower order of magnitude, and the blockchain system cannot be fully utilized. Home-based smart home networks often cover more nodes, providing a suitable environment for blockchain applications. Therefore, the model proposed in this paper is mainly applied to smart home networks with homes as independent nodes (the gateway of the home can be considered as a connect access). In the use case study, each home is considered as a node, communications and remote access between each other are mapped as transactions. In a smart home system the smart home managers can be considered as gateways and managers of homes. Similarly, they are treated as nodes in the home-based blockchain network. These nodes not only store data but also can be miners. The following are the case studies for discussing the advantages of the model:

*Smart Home communication*: Consider device installation as a transaction. When a home installed a smart device, the information of “This home installed this kind of device and has the driver software” is recorded. If another smart home wants to deploy the same kind of device, the first home manager declares that “I have the driver and a communication can be constructed to transfer it.” But before doing transmission, the second home manager must check whether the first smart home has the software through the blockchain. After the transmission, the information of “The first home transferred the device driver to the second home” will be recorded. Different from the original blockchain working mechanism, in this model, only some of the home managers need to participate in the verification and storage process. This working mechanism reduces the storage capacity of the entire network.

*Smart Home access*: Take a device request as a transaction and consider the situation in which Alice invites Bob to her home for a party. When Bob arrives at Alice’s home, the smart access control in Alice’s home gets the entry request and sends the information “Bob is in Alice’s home” to the blockchain. After that Bob wants to check the situation of his own home through Alice home’ network using his cell phone. The access from Alice’s home to Bob’s home must be constructed. But before doing this, Bob’s home manager must check whether it is true that Bob has requested access from Alice’s home. The credibility of Bob’s cell phone will be verified by some of the other smart homes which have recorded the information. After Bob’s smart home manager verified Bob’s cell phone, it will permit access and the new information “Bob has accessed his home from Alice’s home using his cell phone” will be sent to the blockchain network.

## 5. Experiments and Evolution

### 5.1. Storage Efficiency Analysis

**Figure 7:**
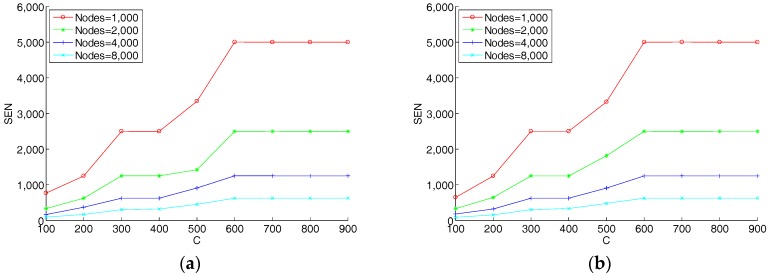

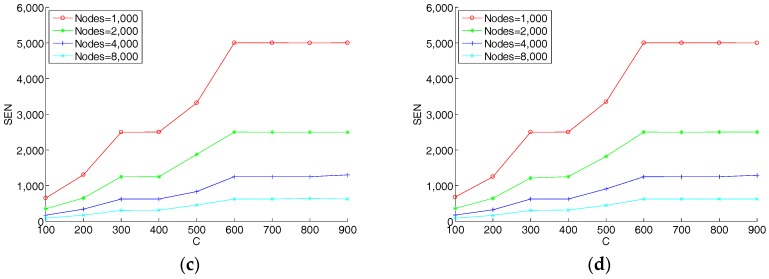
The average storage in each node, (**a**) N = 1, (**b**) N = 2, (**c**) N = 3, and (**d**) N = 4.

In this section we discuss the parameters of the hypergraph-based blockchain network. We study the effect of network parameters on storage efficiency. As discussed in Section 3.3.1, the main parameters are the graph’s degree N and the count of the upper limit of nodes in hyperedge C. These parameters affect the average storage in each node (SEN), or the used memory in each node. The methodology of the designed experiments is that: For a given value of *C* and a number of total nodes in the network we calculate the average storage capacity of nodes and draw the figures. In the following experiments, we assume that the number of transactions in the network is 10^4^. The results are shown in Figure 7. From Figure 7, we can see that with a given N, if the value of C increases, the average record storage capacity of each node continuously increases. The reason is that with the increase of C, the number of nodes in each hyperedge increases, while the number of hyperedges decreases. Each transaction record is stored by more nodes, so that the average storage in each node increases. On the other hand, it also can be seen that the smaller the number of nodes in the graph, the greater the storage capacity of each node. With the same number of transaction records and C values, the smaller the number of nodes, the smaller the corresponding number of hyperedges. Under the premise that the distribution of nodes in each hyperedge is the same, there are more transaction data records in each hyperedge in a graph with fewer hyperedges, resulting in a larger average storage capacity for each node. In particular, when C is equal to the number of nodes in the hypergraph, the proposed model will degenerate into the original blockchain model.

**Figure 8:**
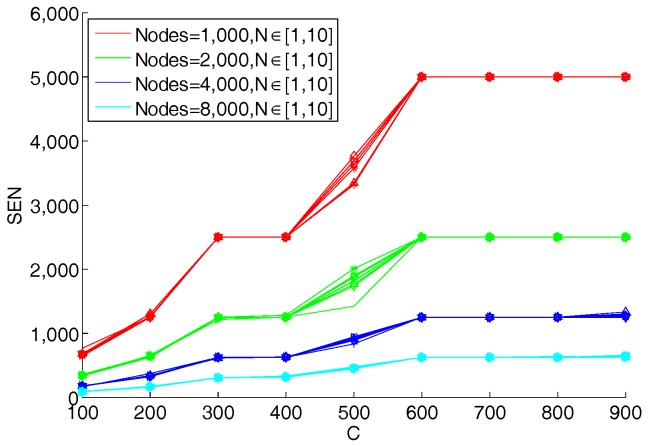
The average storage in each node.

**Figure 9:**
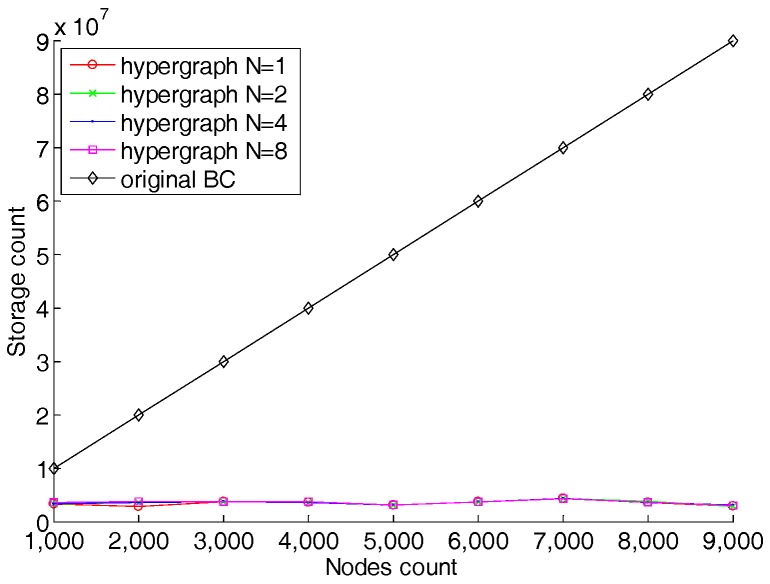
Memory comparison of proposed model with original blockchain model.

Furthermore, we found that for different N, the curves in the images are very similar. Therefore, we superimposed different curves of N from 1 to 10, and the results are shown in Figure 8. It can be seen from Figure 8 that the graph’s degree N of the hypergraph has almost no effect on the average storage of each node. That means the storage of each node is only related to the number of transactions and C. In order to prove the advantage of the hypergraph-based blockchain model, we compared the total used memory capacity in the model with the original blockchain model. The results are shown in Figure 9. The original blockchain stores all records in all nodes, so the used memory capacity for the whole network is in proportion to the number of transactions. It can be clearly seen from Figure 9 that the model proposed in this paper is much smaller than the original blockchain model in terms of overall network storage consumption, and it also shows that the total storage quantity is basically independent with the graph’s degree N.

In the original blockchain model, if 51% of nodes are hacked at the same time, the deception can be established. However, the risk is reduced in the proposed model. Therefore, a bigger C must be set and the arbitration threshold should be set lower.

### 5.2. Network Evolution

The security analysis in Section 3.5 illustrates the importance of the co-rank, which is denoted by C/2 in Section 3.3.1, and the experiments in Section 5.1 show that not only the storage efficiency but also security factors rely on the network parameter C. In this section, we discuss the network evolution with C.

In Section 3.6, we designed a hyperedge splitting algorithm for the evolution of network structure, which affects the average cardinality of the graph. The algorithm guarantees the cardinality of each hyperedge between co-rank(C/2) and rank(C). Therefore, as the number of network nodes increases, the number of network hyperedges will change, but a lower bound and an upper bound will be guaranteed. Let *h_min_(n)* denote the min count of hyperedges in a *n* vertexes hypergraph and *h_max_*(*n*) to the max. Depending on the algorithm, we get the following formulas:

(1) $$\;h_{min\;}\left(n\right)=n/C+1.$$

(2) $$\;h_{max\;}\left(n\right)=n/\frac{C}{2}+1.$$

Equation (1) means that all vertexes always insert into one hyperedge and split the hyperedge. Then for all hyperedges in the graph, there are only C/2 vertexes in each edge. For the Formula (2), vertexes are inserted into the edge with the fewest number of vertexes each time, only when all hyperedges have C nodes does the splitting happen. As a general situation, a new vertex is inserted into a hyperedge randomly. Then the nodes are uniformly distributed, meaning that when there are *k* hyperedges in the graph, after inserting k*C/2 nodes, the k nodes inserted next will cause the *k* edges to split. Thus, if we let *h*(*n*) denote the count of hyperedges, it should meet the following formula:

(3) $$\;h\left(n+1\;\right)=\{\begin{array}{c} h\left(n\right),\;n+1<h\left(n\right)\times C\;\\ h\left(n\right)+1,\;h\left(n\right)\times C<n+1<\left(h\left(n\right)+1\right)\times C. \end{array}$$

**Figure 10:**
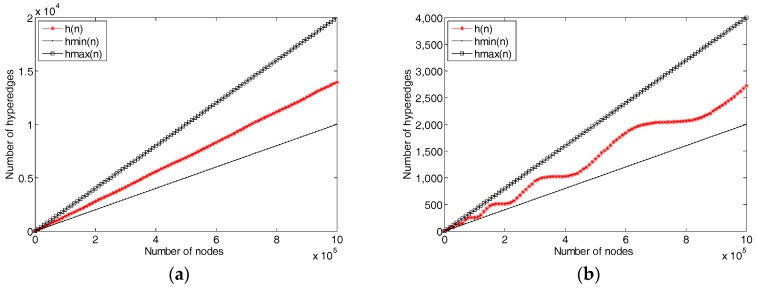

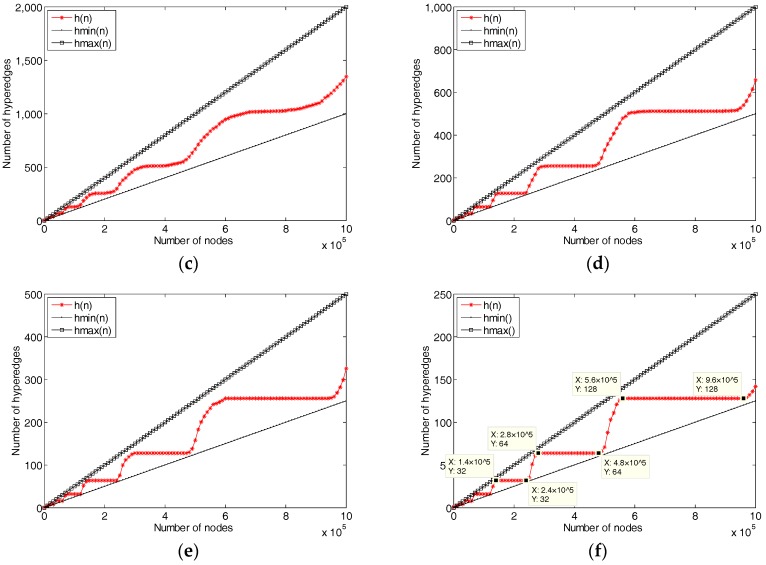
The network evolution with different C, (**a**) C = 100, (**b**) C = 500, (**c**) C = 1,000, (**d**) C = 2000, (**e**) C = 4000, (**f**) C = 8000.

We designed experiments to verify this characteristic of the network, and the results are shown in Figure 10. Figure 10 shows that with the increase of C, the capacity of each hyperedge increases, and the change of hyperedge number is more and more stepwise, which is in line with the description of Equation (3). Particularly, in Figure 10f, the height and length of each step is almost twice that of the previous level, which is consistent with the analysis results.

The experiments in Section 5.1 show that for minimizing the entire network storage capacity, we need a small C, but as discussed in Section 3.5, for security, we need a big C; this is a contradiction. Therefore, we need to find the minimum value of C to maintain security. The problem is that for a given verification threshold *t*, how many forged nodes must be joined in order to construct a hyperedge with a forged node ratio exceeding *t*. Let *n*(*i*) denote the nodes counted in a hypergraph with *i* hyperedges. Based on experiments in Section 5.2, we can approximately consider that:

(4) $$\;n\left(i+1\;\right)=n\left(i\right)+n\left(i\right)\times \frac{C}{2}.$$

Obviously, *n*(1) ∈ [1, *C*/2], and C is much bigger than 1. Therefore, from iteration Formula (4) we get the general term of *n(i*):

(5) $$\;n(i)\;=n\left(1\right)\times {\left(1+\frac{C}{2}\right)}^{i-1}\approx {\left(1+\frac{C}{2}\right)}^{i-1}\approx {\left(\frac{C}{2}\right)}^{i-1}.$$

From *n*(*i*) to *n*(*i* + 1) the new node ratio goes to 50% for each hyperedge, and the count of nodes we added is calculated in Equation (6):

(6) $$\;n\left(i+1\;\right)-n\left(i\right)={\left(\frac{C}{2}\right)}^{i}-{\left(\frac{C}{2}\right)}^{i-1}=\left(\frac{C}{2}-1\right)\times {\left(\frac{C}{2}\right)}^{i-1}.$$

Under equal probability conditions, if we want a hyperedge with a forged node ratio exceeding *t*, we at least add $\log_{2}\frac{1}{1-t}$ edges. The total nodes added, *T*(*n*)*,* is calculated in Equation (7):

(7) $$T(n)\;=n\left(i+{log}_{2}\frac{1}{1-t}\right)-n\left(i\right)={\left(\frac{C}{2}\right)}^{i-1}\times \left({\left(\frac{C}{2}\right)}^{{log}_{2}\frac{1}{1-t}}-1\right).$$

The edge count *i* is related with the node count in the network in Equation (3) and can be approximately considered to be $i=\log_{2}\left(N+1\right)$ (approximate calculation according to Figure 10f) where *N* denotes the count of nodes in the network. Equation (7) becomes to Equation (8):

(8) $$T(n)\;={\left(\frac{C}{2}\right)}^{{log}_{2}\frac{N+1}{2}}\times \left({\left(\frac{C}{2}\right)}^{{log}_{2}\frac{1}{1-t}}-1\right).$$

In Equation (8), *T*(*n*) denotes how difficult it is to forge a hyperedge. It is related with the rank *C*, network scale *N*, and the given verification threshold *t*. For the given parameters of *T*(*n*)*, N* and *t,* we get the smallest *C* by solving Equation (8).

## 6. Conclusions

With the fast development of mobile internet, IoT applications, represented by smart homes, have been applied in many areas. However, IoT applications still face many security and privacy challenges. Blockchain technology, which underpins the crypto-currency bitcoin, has played an important role in the development of decentralized and data intensive applications running on millions of devices. Due to the less energy and memory of most IoT devices, we propose a hypergraph-based blockchain model. By abstracting the blockchain network in a hypergraph, we considered each home as a node in the graph and used hypergraph theory to discount the network’s storage for records. The working mechanism, security risks and response strategy were discussed. The hypergraph based blockchain model proposed in this paper can be applied to smart homes and can facilitate maintaining security and privacy protection requirements. The experiments showed that the storage capacity is better than the original blockchain. The results also show that the average storage in each node increases with its rank in the hyperedge and is independent from the degree of the graph. We studied the network evolution using the proposed algorithm of splitting and aggregation hyperedges. A formula about the security relation to the graph rank and verification threshold was given.

Although the model can improve the storage capacity of blockchain, there are still some issues that need to be further studied. First, the construction of the linearly independency matrix needs an effective algorithm to support it, and if the order is allowed to be changed, the communication protocol must be designed. Second, the accuracy of attack detection needs to be studied. More experiments must be done to adjust network parameters and improve them.

## Figures and Tables

**Table 1:** Comparison of blockchain models.

Model	Storage	Blockchain Structure	Verification	Miners’ Function
Original blockchain	One node one copy	One chain	By node itself	POW
Hypergraph-based blockchain	Part nodes have a copy	Several subchains	By other nodes	POW and linear independence matrix
